# Quantitative evaluation of toothbrush and arm-joint motion during tooth brushing

**DOI:** 10.1007/s00784-014-1367-2

**Published:** 2014-12-04

**Authors:** Emi Inada, Issei Saitoh, Yong Yu, Daisuke Tomiyama, Daisuke Murakami, Yoshihiko Takemoto, Ken Morizono, Tomonori Iwasaki, Yoko Iwase, Youichi Yamasaki

**Affiliations:** 1 Department of Pediatric Dentistry, Kagoshima University Graduate School of Medical and Dental Sciences, 8-35-1 Sakuragaoka, Kagoshima, 890-8544 Japan; 2 Division of Pediatric Dentistry, Graduate School of Medical and Dental Science, Niigata University, 2-5274 Gakkocho-dori, Chuo-ku, Niigata 951-8514 Japan; 3 Department of Mechanical Engineering, Graduate School of Science and Engineering, Kagoshima University, 1-21-4 Korimoto, Kagoshima, 890-0065 Japan

**Keywords:** Tooth brushing, Toothbrush motion, Arm-joint motion, Quantitative motion acquisition, Distinctive rhythm

## Abstract

**Objectives:**

It is very difficult for dental professionals to objectively assess tooth brushing skill of patients, because an obvious index to assess the brushing motion of patients has not been established. The purpose of this study was to quantitatively evaluate toothbrush and arm-joint motion during tooth brushing.

**Materials and methods:**

Tooth brushing motion, performed by dental hygienists for 15 s, was captured using a motion-capture system that continuously calculates the three-dimensional coordinates of object’s motion relative to the floor. The dental hygienists performed the tooth brushing on the buccal and palatal sides of their right and left upper molars. The frequencies and power spectra of toothbrush motion and joint angles of the shoulder, elbow, and wrist were calculated and analyzed statistically.

**Results:**

The frequency of toothbrush motion was higher on the left side (both buccal and palatal areas) than on the right side. There were no significant differences among joint angle frequencies within each brushing area. The inter- and intra-individual variations of the power spectrum of the elbow flexion angle when brushing were smaller than for any of the other angles.

**Conclusions:**

This study quantitatively confirmed that dental hygienists have individual distinctive rhythms during tooth brushing. All arm joints moved synchronously during brushing, and tooth brushing motion was controlled by coordinated movement of the joints. The elbow generated an individual’s frequency through a stabilizing movement.

**Clinical relevance:**

The shoulder and wrist control the hand motion, and the elbow generates the cyclic rhythm during tooth brushing.

## Introduction

Learning tooth brushing is necessary because oral health is one of the most important factors for not only prevention of oral cavity and periodontal diseases but also general health [1, 2]. Dental professionals need to perform appropriate instruction of tooth brushing to patients and clinically monitor their progress of brushing skills. Currently, a plaque-staining agent is the only available method for assessing the effectiveness of plaque removal by tooth brushing. However, this does not allow the dental professional to evaluate the brushing motion used [3, 4] or offer guidance to improve tooth brushing.

In the clinical situation, patients are instructed about tooth brushing motion by leaflets and demonstrations [5] without full recognition of the motion used by the patient [6]. Without visual feedback during instruction, it is also difficult for patients to visualize their own tooth brushing motion based on instructions. Visualization and digitalization of tooth brushing motion would be expected to improve the efficiency of instruction.

Several studies have reported on three-dimensional (3-D) visualization systems for analysis and evaluation of tooth brushing. Kyeong et al. proposed a toothbrush monitoring and training system that senses the user’s brushing pattern by analyzing the wave forms acquired form a built-in accelerometer and magnetic sensor [7]. Graetz et al. proposed digital toothbrush monitoring and training system that could be used to correct brushing motion in the at-home environment. They reported that their visualization system is effective at improving brushing technique and oral hygiene [8]. Although their 3-D visualization system is effective for tooth brushing education and training, it analyzes only toothbrush motion and cannot evaluate the associated arm motion moving the toothbrush.

Daily activities require smoothness and efficiency, with coordinated actions of all parts of the body, for example, coordinated movement of the head, arms, and feet during walking and hopping [9–11]. Tooth brushing motion is a coordinated movement of each arm joint and the mouth. Clarifying how and which parts of the arm coordinate would help improve guidance of tooth brushing. Quantitative evaluations and clear guidance of brushing motion, with both observational and subjective components, will make the instruction and education of brushing skill more efficient. An evidence-based, step-by-step index of brushing skill would make possible standardization of teaching content and methods.

Motion-capture systems can record human motion quantitatively while allowing natural human movement. Such systems have been applied to sports medicine, movies, computer animation, and so on [12, 13]. In this study, we quantitatively recorded the 3-D motion of a toothbrush and arm segments during tooth brushing using a motion-capture system.

We focused on the shoulder, elbow, and wrist motion and the reciprocating motion of the toothbrush during brushing. The frequency and power spectrum of each part’s motion were calculated as a quantitative index. We also evaluated the coordinated movement of each part of the arm and the toothbrush motion that reflected the arm motion biomechanically.

## Materials and methods

### Human subjects

Nine dental hygienists (average age 33 years old; SD 10 years) participated in this study. They were healthy females who worked in the Kagoshima University Medical and Dental Hospital. All subjects were right-handed and free from pain and dysfunction in the craniomandibular, neck, trunk, and limb regions. Informed consent to participate in this study was obtained from all subjects. Informed consent was obtained from the subjects according to the Helsinki Declaration prior to their entering the study. This study was approved by the clinical ethics committee of Kagoshima University Hospital (No. 102).

### Capture system

**Fig. 1:**
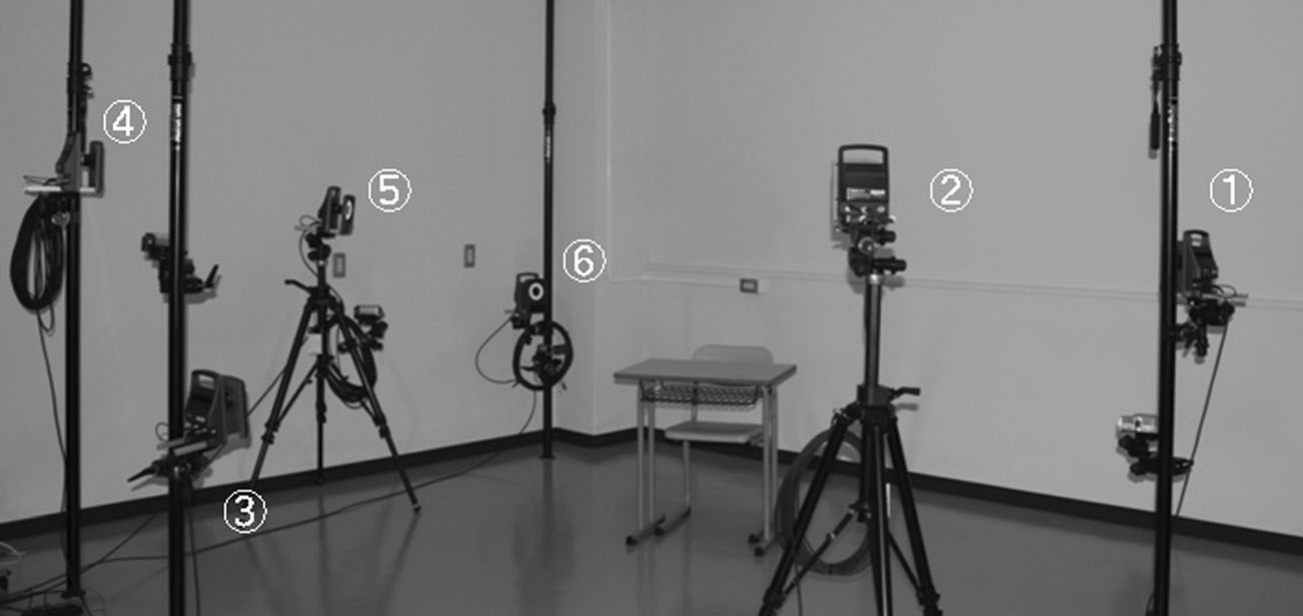
Motion-capture system

**Fig. 2:**
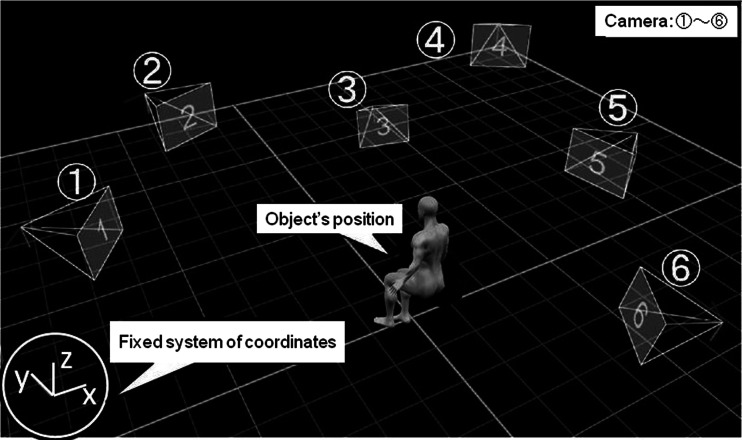
View of camera setup

The Vicon system^Ⓡ^ (Inter-Reha Co. Ltd., Tokyo, Japan) was used as an optical 3-D motion analyzer. Light-reflective balls were attached to the skin of each subject, and these markers’ motion was tracked with six charge-coupled device (CCD) cameras and visualized on a computer display (Fig. 1). Six CCD cameras were placed 1 m away from each subject, with four cameras (1, 2, 4, 5) placed at a height of 1 m, and the remaining cameras (3, 6) placed at a height of 0.5 m (Fig. 2). The markers’ 3-D coordinates were tabulated at 100 Hz.

The subjects were seated comfortably in an upright position with back support up to the mid-scapular level but without a headrest. The subjects were instructed to stare at a mark at eye level in front of them in order to keep a natural head position. Spherical low-weight retro-reflective markers were attached to the body, right arm, and the shaft of toothbrush (see below).

### Marker configuration and test procedure

**Fig. 3:**
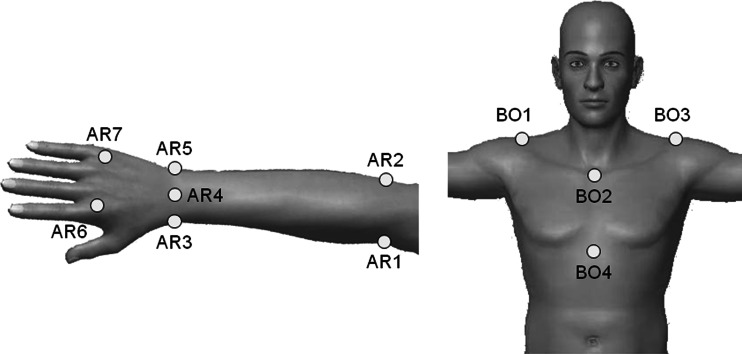
Markers attached to the body, arm, and hand

**Fig. 4:**
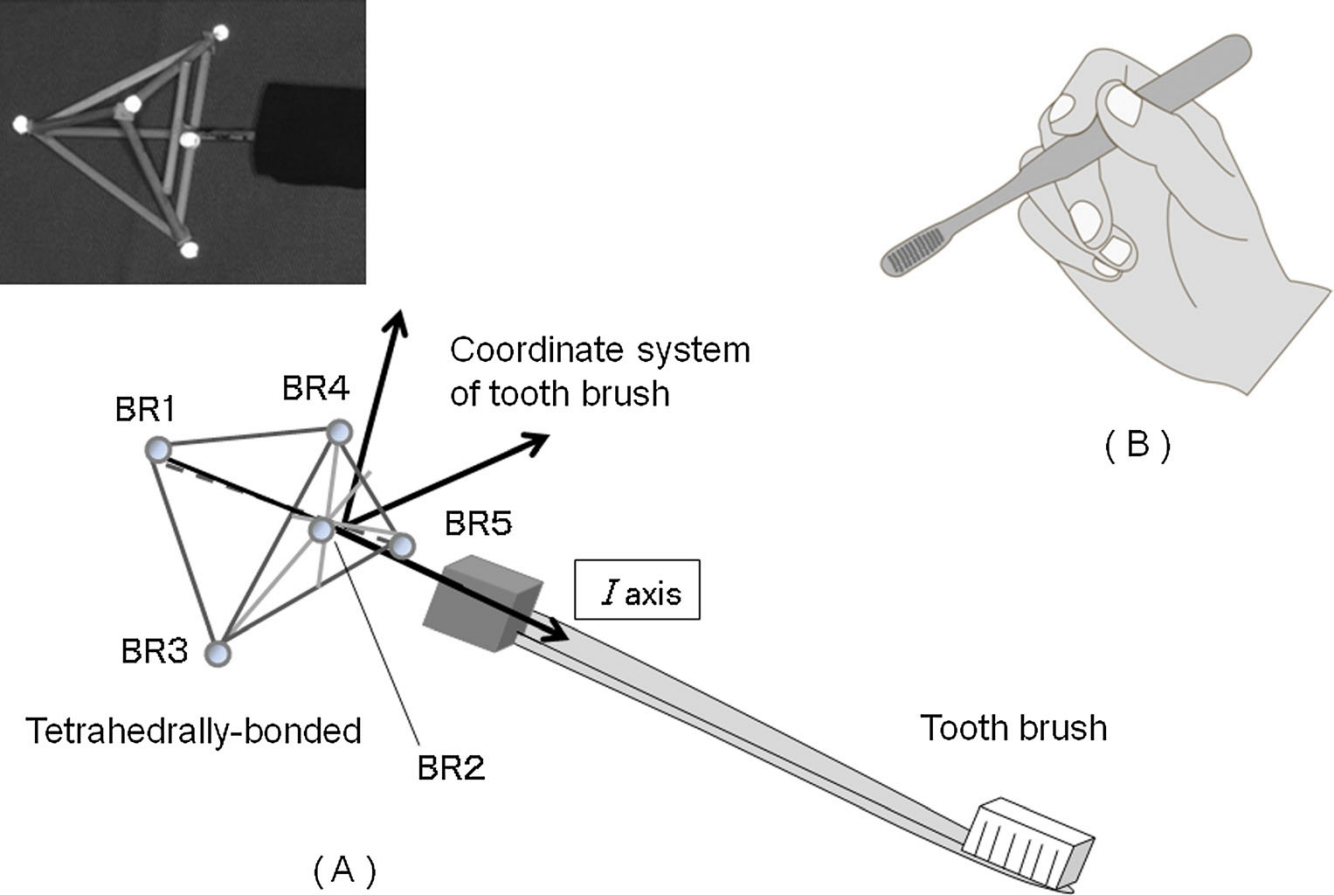
**a** Toothbrush attached to the tetrahedrally bonded markers and its coordinate system. **b** Pen grip

Retro-reflective markers (9 mm in diameter) were attached to the following: (1) the jugular notch of the sternum (BO2), xiphoid process of the sternum (BO4), and the right and left acromion processes (BO1, BO3) to record upper body motion and (2) the inner and outer elbow (AR1, AR2) and wrist (AR3, AR5), the back of wrist (AR4), and dorsum of the hand (AR6, AR7) of the right arm to record arm motion (Fig. 3). In addition, five retro-reflective markers (3.5 mm in diameter) were tetrahedrally bonded (BR1-BR5) and attached to the toothbrush to record its motion (Fig. 4a).

**Fig. 5:**
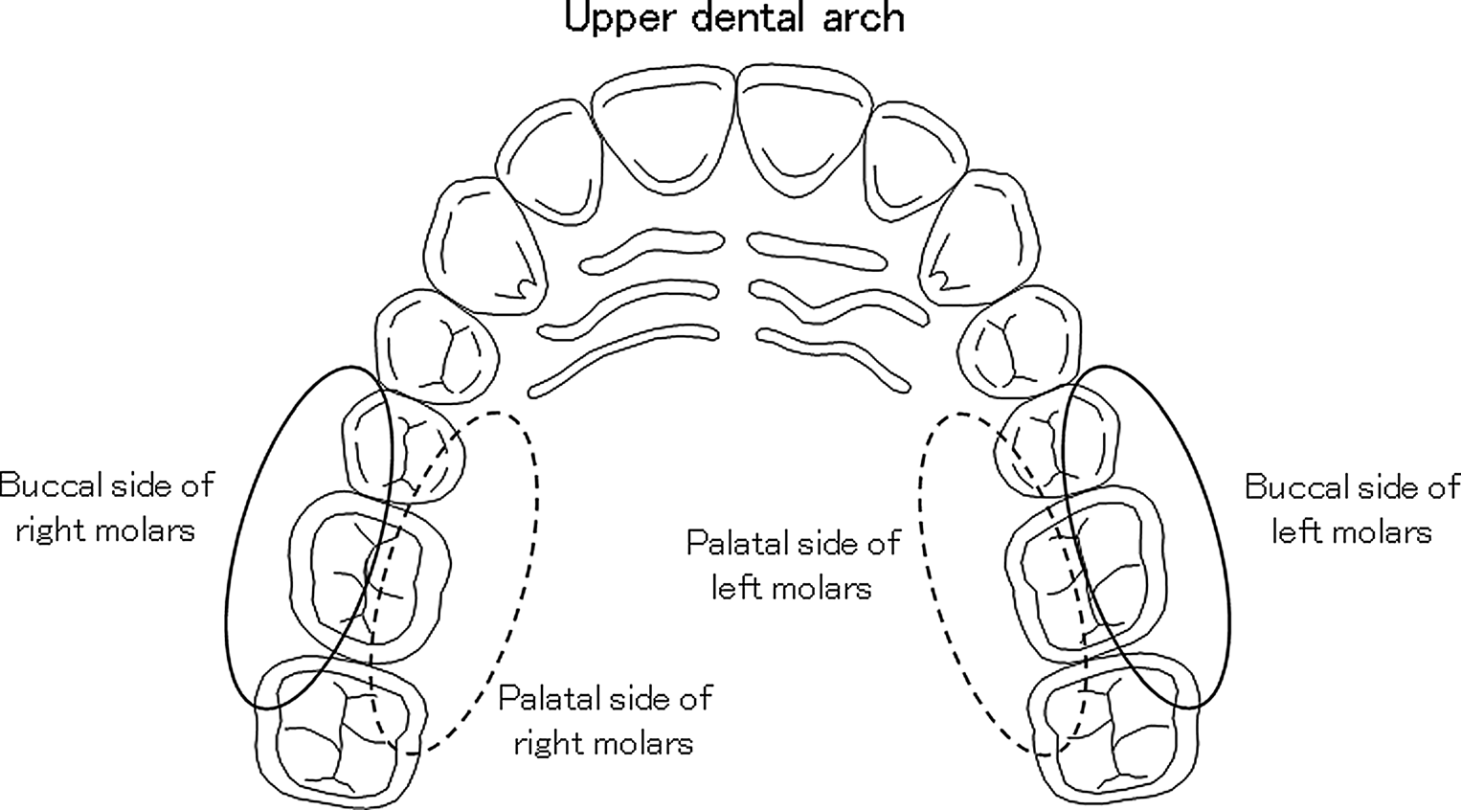
Brushing area of this study

In this study, we focused on the shoulder, elbow, and wrist motion and the reciprocating motion of the toothbrush during brushing. Subjects were instructed to hold the toothbrush with a pen grip (Fig. 4b), which is popular in Japan. The brushing technique (e.g., Scrub and Bass, etc.) was not specified; subjects were only instructed to brush as usual. Brushing was confined to the buccal and palatal sides of the right and left upper molars (Fig. 5), and brushing was repeated three times in each area for 15 s each.

### Data preparation

The 3-D coordinates of each marker’s position relative to the floor were recorded at 100 samples/s.

**Fig. 6:**
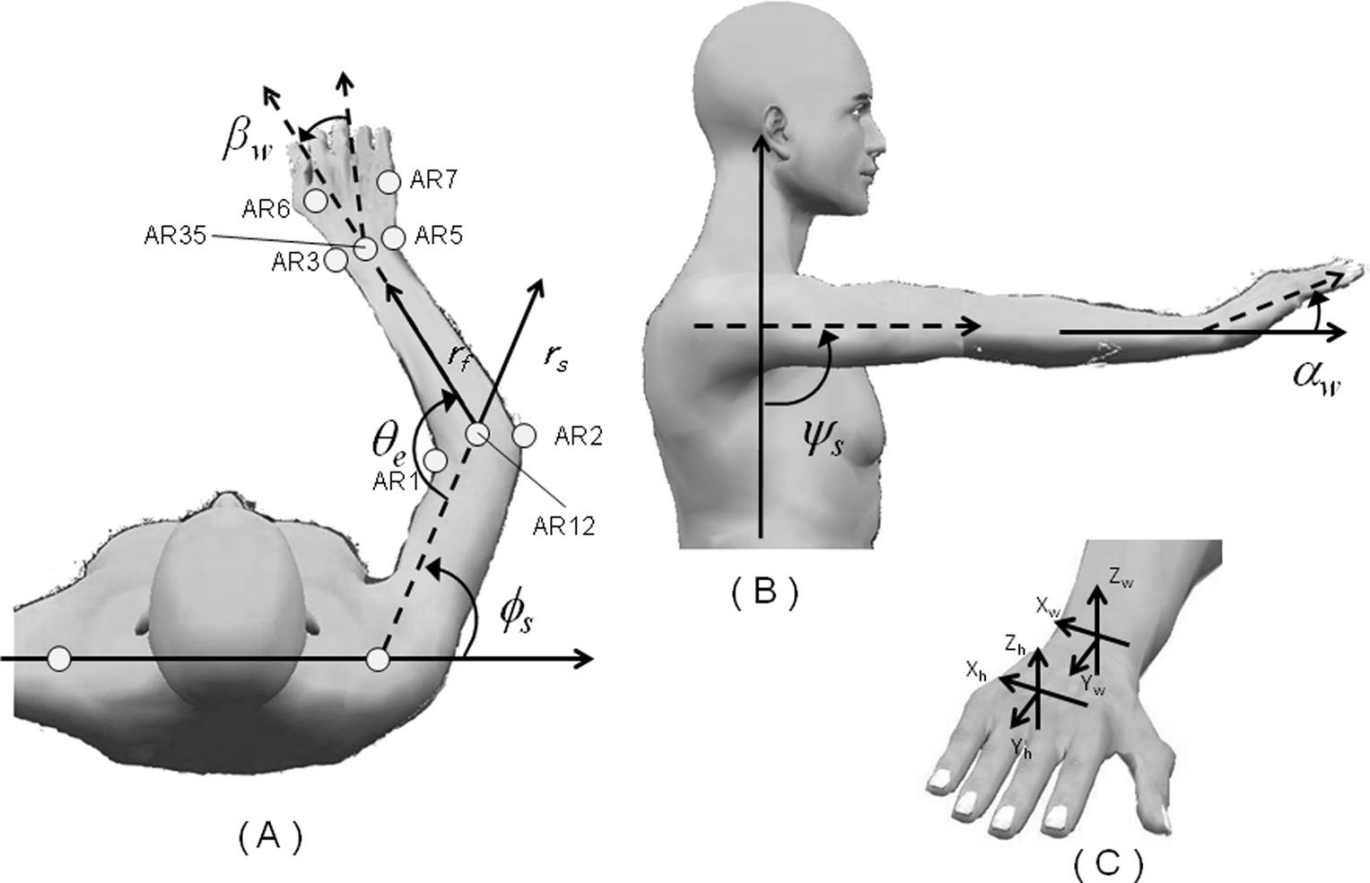
Angles of the shoulder, elbow, and wrist

1. Reciprocating motion of the toothbrush at each area
  Toothbrush motion during brushing was a reciprocating motion parallel to the dental arch. However, brushing was accompanied by a position change of the toothbrush. To analyze only the reciprocating motion of the toothbrush, we set a new coordinate system for the toothbrush, thus eliminating the influence of the position change.
  In the new coordinate system, the axis from BR1 to BR2 was termed the *I*-axis, with its origin at BR2 (Fig. 4a). Unit vectors along the *I*-axis (*I*_*ei*_) at time *i*[1/100 s] were calculated according to the following equation, where BR*n*_*xi*_, BR*n*_*yi*_, and BR*n*_*zi*_ were the *x*, *y*, and *z* coordinates of BR*n* at time *i* and *n* = 1, 2.
  $$ {I}_{ei}=\frac{\left(\begin{array}{c}\hfill \mathrm{B}\mathrm{R}{2}_{xi}\hbox{-} \mathrm{B}\mathrm{R}{1}_{xi}\hfill \\ {}\hfill \mathrm{B}\mathrm{R}{2}_{yi}\hbox{-} \mathrm{B}\mathrm{R}{1}_{yi}\hfill \\ {}\hfill \mathrm{B}\mathrm{R}{2}_{zi}\hbox{-} \mathrm{B}\mathrm{R}{1}_{zi}\hfill \end{array}\right)}{\left\Vert \begin{array}{c}\hfill \mathrm{B}\mathrm{R}{2}_{xi}\hbox{-} \mathrm{B}\mathrm{R}{1}_{xi}\hfill \\ {}\hfill \mathrm{B}\mathrm{R}{2}_{yi}\hbox{-} \mathrm{B}\mathrm{R}{1}_{yi}\hfill \\ {}\hfill \mathrm{B}\mathrm{R}{2}_{zi}\hbox{-} \mathrm{B}\mathrm{R}{1}_{zi}\hfill \end{array}\right\Vert } $$
  The displacement vectors of BR2 (Δ*L*_*i*_) and the *I*-axis translator displacement vectors (Δ*L*_*Ii*_) at time *i*[1/100 s] were calculated according to the following equations.
  $$ \varDelta {L}_i=\left(\begin{array}{c}\hfill \mathrm{B}\mathrm{R}{2}_{x\left(i+1\right)}-\mathrm{B}\mathrm{R}{2}_{xi}\hfill \\ {}\hfill \mathrm{B}\mathrm{R}{2}_{y\left(i+1\right)}-\mathrm{B}\mathrm{R}{2}_{yi}\hfill \\ {}\hfill \mathrm{B}\mathrm{R}{2}_{z\left(i+1\right)}-\mathrm{B}\mathrm{R}{2}_{zi}\hfill \end{array}\right) $$
  $$ \varDelta {L}_{Ii}=\varDelta {L}_i\cdot {I}_{ei} $$
  Thus, the reciprocating motion of the toothbrush could be calculated along the *I*-axis.
2. Motion of the shoulder
  The shoulder has five degrees of freedom, consisting of elevation/depression and flexion/extension of the scapula and flexion/extension, adduction/abduction, and outer/inner rotation of the glenohumeral joint. We focused on the glenohumeral rotation angles of adduction/abduction (*ϕ*_*s*_, Fig. 6) and flexion/extension (*ψ*_*s*_, Fig. 6) because these upper arm movements have a great influence on brushing motion. We constructed a new coordinate system using BO1, BO2, BO3, and BO4. The vector of the upper arm (*r*_*s*_) was formed by BO1 and the midpoint between AR1 and AR2 (AR12). Rotation angles of the upper arm (*ϕ*_*si*_, *ψ*_*si*_) at time *i*[1/100 s] were calculated according to the following equations, where BO*n*_*xi*_, BO*n*_*yi*_, and BO*n*_*zi*_ were the *x*, *y*, and *z* coordinates of BO*n* at time *i*, and where *n* = 1, 2, 3, 4, and with AR12_*xi*_, AR12_*yi*_, and AR12_*zi*_ being the *x*, *y*, and *z* coordinates of AR12.

  $$ {\phi}_{s\ i}={ \cos}^{-1}\frac{{\left(\begin{array}{c}\hfill \mathrm{A}\mathrm{R}{12}_{xi}-\mathrm{BO}{1}_{xi}\hfill \\ {}\hfill \mathrm{A}\mathrm{R}{12}_{yi}-\mathrm{BO}{1}_{yi}\hfill \\ {}\hfill 0\hfill \end{array}\right)}^{\mathrm{T}}\cdot \left(\begin{array}{c}\hfill \mathrm{BO}{1}_{xi}-\mathrm{BO}{3}_{xi}\hfill \\ {}\hfill \mathrm{BO}{1}_{yi}-\mathrm{BO}{3}_{yi}\hfill \\ {}\hfill \mathrm{BO}{1}_{zi}-\mathrm{BO}{3}_{zi}\hfill \end{array}\right)}{\left\Vert \begin{array}{c}\hfill \mathrm{A}\mathrm{R}{12}_{xi}-\mathrm{BO}{1}_{xi}\hfill \\ {}\hfill \mathrm{A}\mathrm{R}{12}_{yi}-\mathrm{BO}{1}_{yi}\hfill \\ {}\hfill 0\hfill \end{array}\right\Vert \cdot \left\Vert \begin{array}{c}\hfill \mathrm{BO}{1}_{xi}-\mathrm{BO}{3}_{xi}\hfill \\ {}\hfill \mathrm{BO}{1}_{yi}-\mathrm{BO}{3}_{yi}\hfill \\ {}\hfill \mathrm{BO}{1}_{zi}-\mathrm{BO}{3}_{zi}\hfill \end{array}\right\Vert } $$
  $$ {\psi}_{s\ i}={ \cos}^{-1}\frac{{\left(\begin{array}{c}\hfill 0\hfill \\ {}\hfill \mathrm{A}\mathrm{R}{12}_{yi}-\mathrm{BO}{1}_{yi}\hfill \\ {}\hfill \mathrm{A}\mathrm{R}{12}_{zi}-\mathrm{BO}{1}_{zi}\hfill \end{array}\right)}^{\mathrm{T}}\cdot \left(\begin{array}{c}\hfill \mathrm{BO}{4}_{xi}-\mathrm{BO}{2}_{xi}\hfill \\ {}\hfill \mathrm{BO}{4}_{yi}-\mathrm{BO}{2}_{yi}\hfill \\ {}\hfill \mathrm{BO}{4}_{zi}-\mathrm{BO}{2}_{zi}\hfill \end{array}\right)}{\left\Vert \begin{array}{c}\hfill 0\hfill \\ {}\hfill \mathrm{A}\mathrm{R}{12}_{yi}-\mathrm{BO}{1}_{yi}\hfill \\ {}\hfill \mathrm{A}\mathrm{R}{12}_{zi}-\mathrm{BO}{1}_{zi}\hfill \end{array}\right\Vert \cdot \left\Vert \begin{array}{c}\hfill \mathrm{BO}{4}_{xi}-\mathrm{BO}{2}_{xi}\hfill \\ {}\hfill \mathrm{BO}{4}_{yi}-\mathrm{BO}{2}_{yi}\hfill \\ {}\hfill \mathrm{BO}{4}_{zi}-\mathrm{BO}{2}_{zi}\hfill \end{array}\right\Vert } $$
3. Motion of the elbow
  The elbow has a single degree of freedom of rotation, i.e., flexion/extension of the joint of elbow. The vector of the forearm (*r*_*f*_, Fig. 6) was formed by point AR12 and the midpoint between AR3 and AR5 (AR35). Rotation angles of the elbow (*θ*_*ei*_) at time *i*[1/100 s] were calculated by the following equation, where AR35_*xi*_, AR35_*yi*_, and AR35_*zi*_ are the *x*, *y*, and *z* coordinates of AR35.
  $$ {\theta}_{e\ i} = { \cos}^{-1}\frac{{\left(\begin{array}{c}\hfill \mathrm{BO}{1}_{xi}-\mathrm{A}\mathrm{R}{12}_{xi}\hfill \\ {}\hfill \mathrm{BO}{1}_{yi}-\mathrm{A}\mathrm{R}{12}_{yi}\hfill \\ {}\hfill \mathrm{BO}{1}_{zi}-\mathrm{A}\mathrm{R}{12}_{zi}\hfill \end{array}\right)}^{\mathrm{T}}\cdot \left(\begin{array}{c}\hfill \mathrm{A}\mathrm{R}{35}_{xi}-\mathrm{A}\mathrm{R}{12}_{xi}\hfill \\ {}\hfill \mathrm{A}\mathrm{R}{35}_{yi}-\mathrm{A}\mathrm{R}{12}_{yi}\hfill \\ {}\hfill \mathrm{A}\mathrm{R}{35}_{zi}-\mathrm{A}\mathrm{R}{12}_{zi}\hfill \end{array}\right)}{\left\Vert \begin{array}{c}\hfill \mathrm{BO}{1}_{xi}-\mathrm{A}\mathrm{R}{12}_{xi}\hfill \\ {}\hfill \mathrm{BO}{1}_{yi}-\mathrm{A}\mathrm{R}{12}_{yi}\hfill \\ {}\hfill \mathrm{BO}{1}_{zi}-\mathrm{A}\mathrm{R}{12}_{zi}\hfill \end{array}\right\Vert \cdot \left\Vert \begin{array}{c}\hfill \mathrm{A}\mathrm{R}{35}_{xi}-\mathrm{A}\mathrm{R}{12}_{xi}\hfill \\ {}\hfill \mathrm{A}\mathrm{R}{35}_{yi}-\mathrm{A}\mathrm{R}{12}_{yi}\hfill \\ {}\hfill \mathrm{A}\mathrm{R}{35}_{zi}-\mathrm{A}\mathrm{R}{12}_{zi}\hfill \end{array}\right\Vert } $$
4. Motion of the wrist
  The wrist has two degrees of freedom of movement, consisting of flexion/extension and outer/inner rotation of the wrist joint. The rotation angle for flexion/extension (*α*_*w*_) and the angle for outer/inner rotation (*β*_*w*_) were calculated from relative angles between the standard plane of the wrist and the back of the hand (Fig. 6). Each coordinate axis for the standard plane of the wrist was calculated according to the following equations.
  $$ {X}_w=\frac{\left(\begin{array}{c}\hfill \mathrm{A}\mathrm{R}{5}_{xi}-\mathrm{A}\mathrm{R}{3}_{xi}\hfill \\ {}\hfill \mathrm{A}\mathrm{R}{5}_{yi}-\mathrm{A}\mathrm{R}{3}_{yi}\hfill \\ {}\hfill \mathrm{A}\mathrm{R}{5}_{zi}-\mathrm{A}\mathrm{R}{3}_{zi}\hfill \end{array}\right)}{\left\Vert \begin{array}{c}\hfill \mathrm{A}\mathrm{R}{5}_{xi}-\mathrm{A}\mathrm{R}{3}_{xi}\hfill \\ {}\hfill \mathrm{A}\mathrm{R}{5}_{yi}-\mathrm{A}\mathrm{R}{3}_{yi}\hfill \\ {}\hfill \mathrm{A}\mathrm{R}{5}_{zi}-\mathrm{A}\mathrm{R}{3}_{zi}\hfill \end{array}\right\Vert } $$
  $$ {Y}_w={Z}_w\times {X}_w $$
  $$ {Z}_{\mathrm{w}}=\frac{\left(\begin{array}{c}\hfill \mathrm{A}\mathrm{R}{4}_{xi}-\mathrm{A}\mathrm{R}{35}_{xi}\hfill \\ {}\hfill \mathrm{A}\mathrm{R}{4}_{yi}-\mathrm{A}\mathrm{R}{35}_{yi}\hfill \\ {}\hfill \mathrm{A}\mathrm{R}{4}_{zi}-\mathrm{A}\mathrm{R}{35}_{zi}\hfill \end{array}\right)}{\left\Vert \begin{array}{c}\hfill \mathrm{A}\mathrm{R}{4}_{xi}-\mathrm{A}\mathrm{R}{35}_{xi}\hfill \\ {}\hfill \mathrm{A}\mathrm{R}{4}_{yi}-\mathrm{A}\mathrm{R}{35}_{yi}\hfill \\ {}\hfill \mathrm{A}\mathrm{R}{4}_{zi}-\mathrm{A}\mathrm{R}{35}_{zi}\hfill \end{array}\right\Vert } $$
  Each coordinate axis of the standard plane of the back of the hand was calculated according to the following equations, where AR67_*xi*_, AR67_*yi*_, and AR67_*zi*_ are the *x*, *y*, and *z* coordinates of the midpoint of AR6 and AR7 (AR67).
  $$ {X}_h=\frac{\left(\begin{array}{c}\hfill \mathrm{A}\mathrm{R}{7}_{xi}-\mathrm{A}\mathrm{R}{6}_{xi}\hfill \\ {}\hfill \mathrm{A}\mathrm{R}{7}_{yi}-\mathrm{A}\mathrm{R}{6}_{yi}\hfill \\ {}\hfill \mathrm{A}\mathrm{R}{7}_{zi}-\mathrm{A}\mathrm{R}{6}_{zi}\hfill \end{array}\right)}{\left\Vert \begin{array}{c}\hfill \mathrm{A}\mathrm{R}{7}_{xi}-\mathrm{A}\mathrm{R}{6}_{xi}\hfill \\ {}\hfill \mathrm{A}\mathrm{R}{7}_{yi}-\mathrm{A}\mathrm{R}{6}_{yi}\hfill \\ {}\hfill \mathrm{A}\mathrm{R}{7}_{zi}-\mathrm{A}\mathrm{R}{6}_{zi}\hfill \end{array}\right\Vert } $$
  $$ {Y}_w=\frac{\left(\begin{array}{c}\hfill \mathrm{A}\mathrm{R}{67}_{xi}-\mathrm{A}\mathrm{R}{4}_{xi}\hfill \\ {}\hfill \mathrm{A}\mathrm{R}{67}_{yi}-\mathrm{A}\mathrm{R}{4}_{yi}\hfill \\ {}\hfill \mathrm{A}\mathrm{R}{67}_{zi}-\mathrm{A}\mathrm{R}{4}_{zi}\hfill \end{array}\right)}{\left\Vert \begin{array}{c}\hfill \mathrm{A}\mathrm{R}{67}_{xi}-\mathrm{A}\mathrm{R}{4}_{xi}\hfill \\ {}\hfill \mathrm{A}\mathrm{R}{67}_{yi}-\mathrm{A}\mathrm{R}{4}_{yi}\hfill \\ {}\hfill \mathrm{A}\mathrm{R}{67}_{zi}-\mathrm{A}\mathrm{R}{4}_{zi}\hfill \end{array}\right\Vert } $$
  $$ {Z}_h={X}_h\times {Y}_h $$
  Rotation angles of the wrist (*α*_*wi*_, *β*_*wi*_) at time *i*[1/100 s] were calculated according to the following equations.
  $$ {\alpha}_{wi}={ \cos}^{-1}\frac{\left({Z}_w\cdot {Z}_h\right)}{\left\Vert {Z}_w\cdot {Z}_h\right\Vert } $$
  $$ {\beta}_{wi}={ \cos}^{-1}\frac{\left({X}_w\cdot {X}_h\right)}{\left\Vert {X}_w\cdot {X}_h\right\Vert } $$

### Data analysis

Brushing motion is one of many human cyclic motions. Mastication, soft plate action during food transport, and head motion during walking are cyclic motions, and they are frequently expressed and analyzed as a frequency [14–17]. A graph of the converted data showing the strength of each independent component is termed a power spectrum. Human cyclic motion is composed of a mixture of several kinds of motion occurring at different frequencies. The most common frequency is detected by plotting the generation rate of each mixed frequency into a graph. The most common frequency becomes the frequency of motion targeted for the analysis. Moreover, the power spectrum correlates with the energy of movement at the appropriate frequency. The power spectrum is effective in quantitatively analyzing cyclic motion that has a mixed number of rhythms (i.e., frequencies and energy).

In this study, the amount of displacement of the toothbrush and the angle change of each joint during brushing were converted to a power spectrum by Fourier transformation, and the frequency range containing most of the power was selected. The frequency of the toothbrush motion may be a quantitative index of the rhythm of tooth brushing motion. In addition, the frequencies and power spectra of the joint angles may be helpful in the understanding of the coordination and role of each joint during brushing.

### Statics

The frequency and power spectrum of the toothbrush motion and joint angles of the shoulder, elbow, and wrist were calculated for each subject during brushing of the buccal and palatal sides of the right and left upper molars. The data were evaluated for right and left side differences for each buccal and palatal area using the Wilcoxon signed-rank test. To compare each side and each area, cross-joint comparisons of the frequency and power spectrum were evaluated using the Friedman test and the Wilcoxon signed-rank test. To test for intra- and inter-individual differences of the frequency and power spectrum for each side and each area, multilevel linear models were used (MLwiN® software, University of Bristol).

## Results

**Fig. 7:**
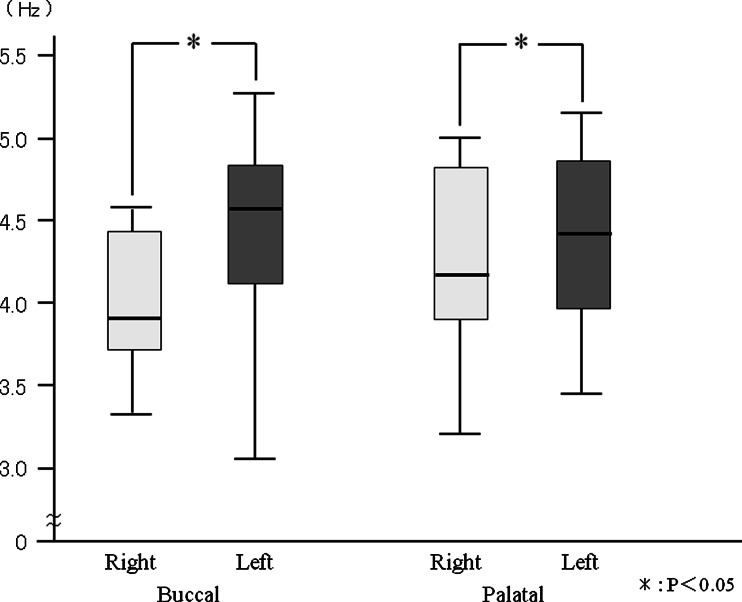
The comparison of frequency during tooth brushing

**Fig. 8:**
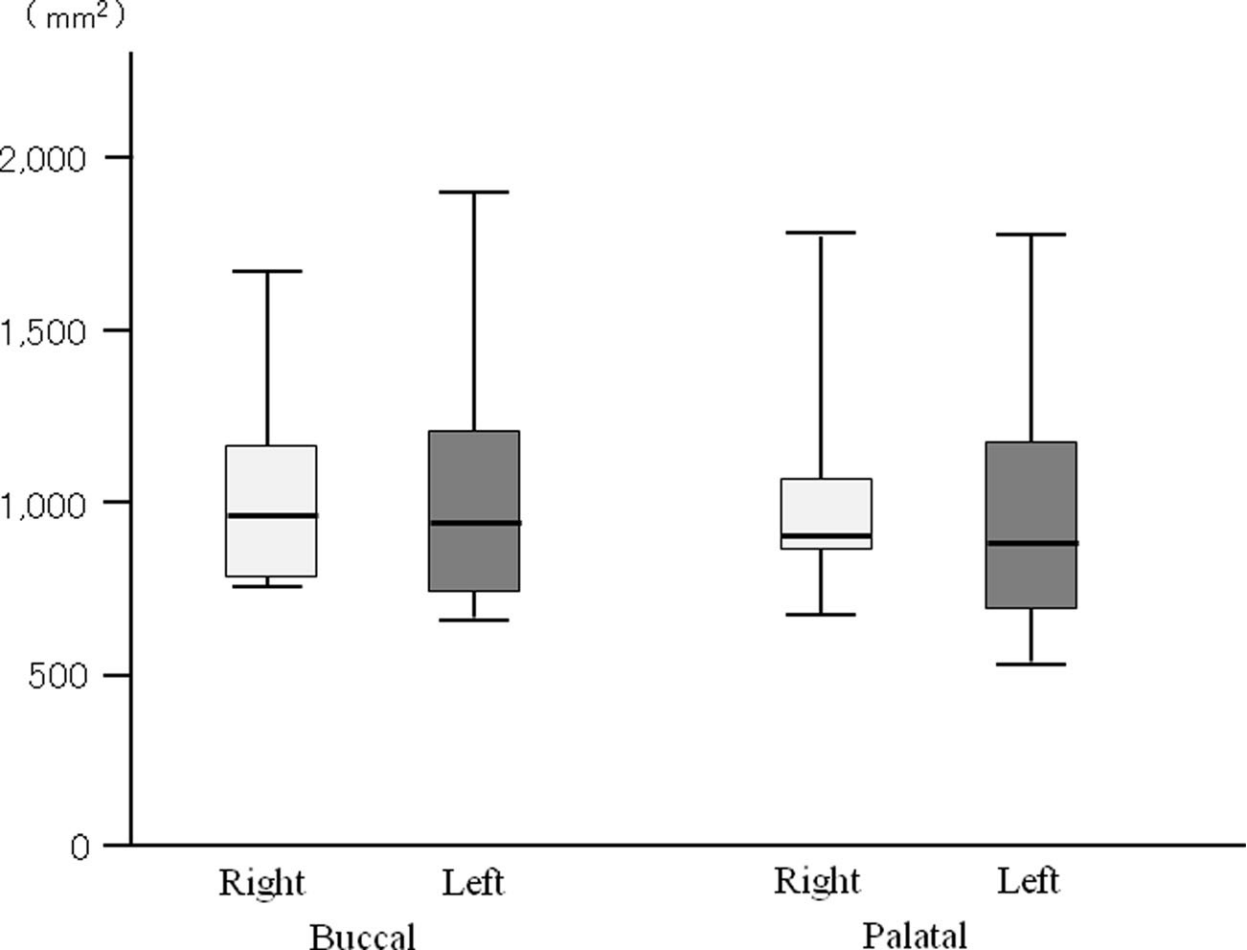
The comparison of power spectrum during tooth brushing

The toothbrush motion frequency was 3.91 Hz on the right buccal side and was 4.56 Hz on the left buccal side. Toothbrush motion frequency was 4.20 Hz on right palatal side and was 4.36 Hz on the left palatal side. The frequency of toothbrush motion was higher on the left side (both buccal and palatal areas) than on the right side (Fig. 7). However, there were no significant side differences in the power spectrum (Fig. 8).

Inter- and intra-individual variations of the frequency and power spectrum of toothbrush motion are shown in Table 1. Intra-individual variation of frequency was smaller than inter-individual variation on both areas and sides. During right side buccal brushing, intra-individual variation of the power spectrum was larger than inter-individual variation, but during left side buccal brushing, the inter-individual variation of the power spectrum was larger. Interestingly, the opposite pattern of variances was seen during palatal brushing.

**Table 1:** Inter- and intra-individual variation of the frequency and power spectrum of toothbrush motion during molar brushing

			Intra-individual	Inter-individual
Hz	Buccal	Right	0.04	0.17
		Left	0.04	0.38
	Palatal	Right	0.02	0.31
		Left	0.03	0.28
PS (×10^4^)	Buccal	Right	10.91	4.26
		Left	2.74	24.31
	Palatal	Right	4.70	16.12
		Left	18.26	7.13

**Fig. 9:**
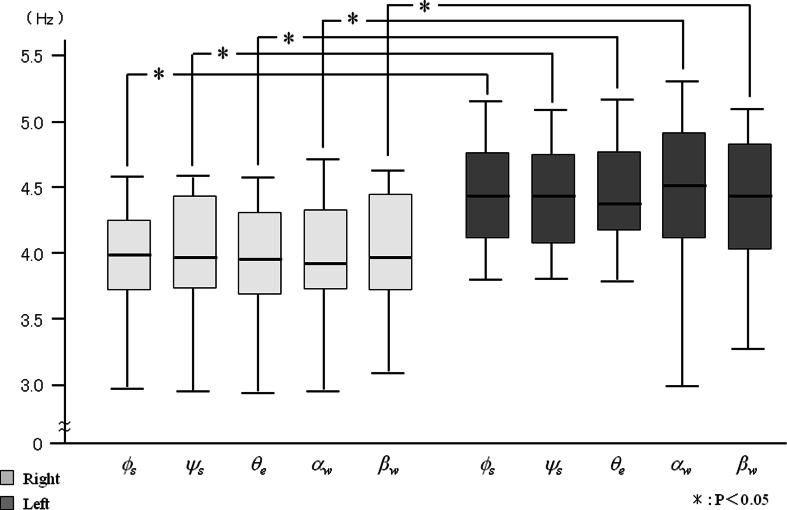
The comparison of frequency of the shoulder, elbow, and wrist angle of buccal brushing

**Fig. 10:**
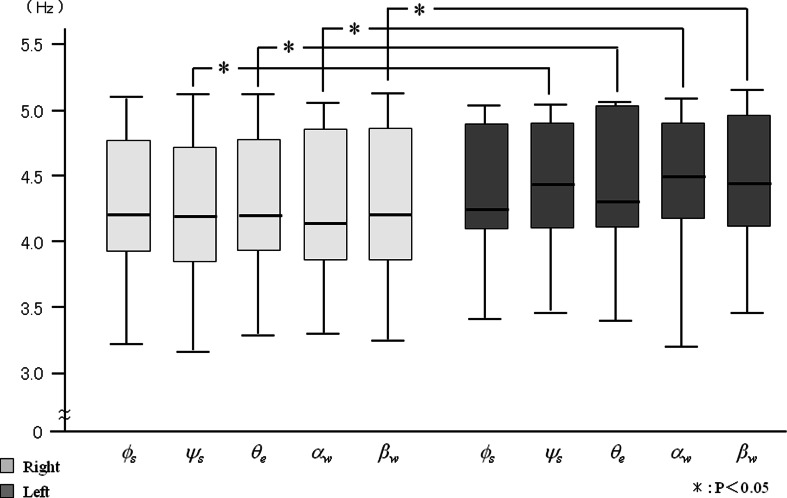
The comparison of frequency of the shoulder, elbow, and wrist angle of palatal brushing

**Fig. 11:**
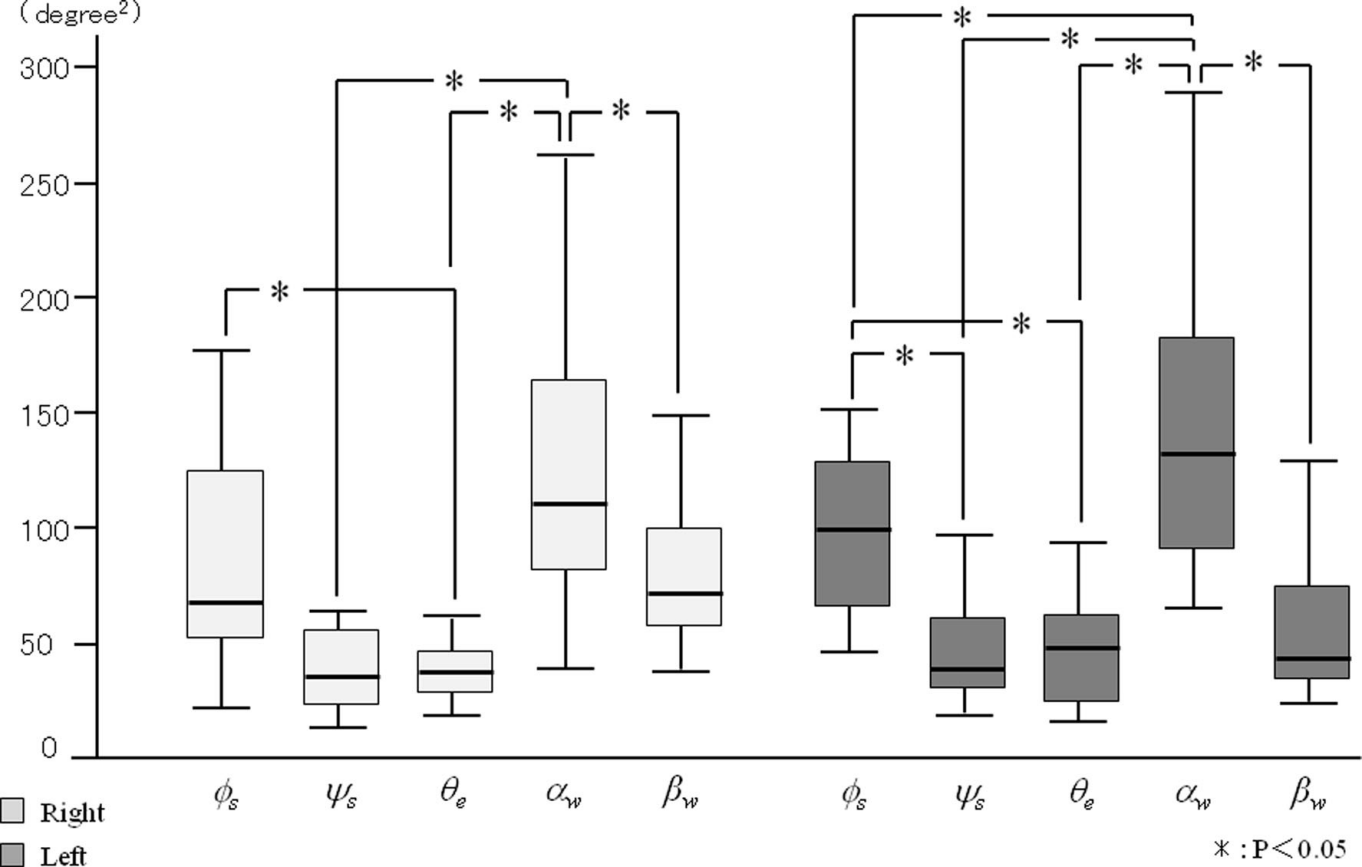
The power spectrum of the shoulder, elbow, and wrist angle of buccal brushing

**Fig. 12:**
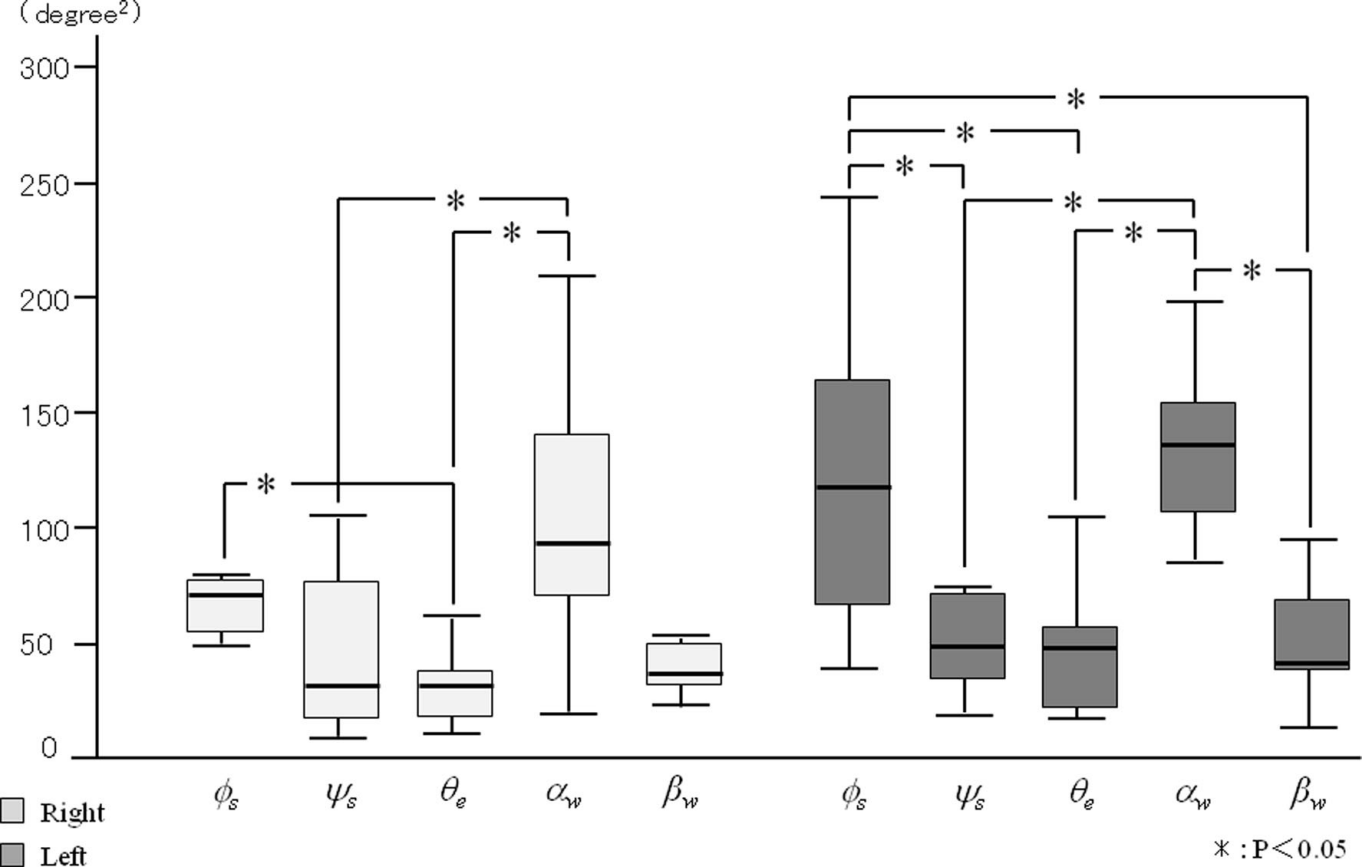
The power spectrum of the shoulder, elbow, and wrist angle of palatal brushing

The frequencies of all joint angles were significantly greater during right side brushing than during left side brushing, except for *ϕ*_*s*_ when brushing the palatal area (Figs. 9 and 10). However, for each side, there were no significant differences in the frequency among the joint angles. In contrast, no significant differences were detected for the power spectrum of joint angles between right and left side brushing for each buccal and palatal area (Figs. 11 and 12). Some joint angles’ power spectra did differ significantly from each other when brushing some palatal and buccal areas of each side.

Inter- and intra-individual variations of the frequencies of the joint angles during brushing are shown in Table 2. Intra-individual variation was smaller than inter-individual variation for all joints under all conditions.

**Table 2:** Inter- and intra-individual variations of joint angle frequency during molar brushing

		Buccal			Palatal		
	Angle	Estimate (SE)	Intra-individual	Inter-individual	Estimate (SE)	Intra-individual	Inter-individual
Right	*ϕ*_*s*_	3.93 (0.15)	0.07	0.18	4.21 (0.19)	0.03	0.31
	*ψ*_*s*_	3.94 (0.16)	0.08	0.20	4.18 (0.19)	0.03	0.32
	*θ*_*e*_	3.96 (0.16)	0.06	0.20	4.22 (0.18)	0.03	0.30
	*α*_*w*_	3.98 (0.15)	0.04	0.18	4.21 (0.19)	0.03	0.30
	*β*_*w*_	3.98 (0.16)	0.08	0.20	4.24 (0.20)	0.05	0.34
Left	*ϕ*_*s*_	4.36 (0.20)	0.02	0.35	4.33 (0.18)	0.02	0.27
	*ψ*_*s*_	4.33 (0.20)	0.02	0.33	4.36 (0.17)	0.02	0.26
	*θ*_*e*_	4.35 (0.20)	0.02	0.35	4.37 (0.18)	0.03	0.28
	*α*_*w*_	4.37 (0.19)	0.02	0.32	4.38 (0.19)	0.03	0.31
	*β*_*w*_	4.35 (0.18)	0.08	0.27	4.43 (0.17)	0.02	0.25

Inter- and intra-individual variations of the power spectrum of the joint angles during brushing are shown in Table 3. When brushing the right side buccal area, intra-individual variations of the vertical shoulder angle (*ψ*_*s*_) and the elbow flexion angle (*θ*_*e*_) were smaller than inter-individual variances, and inter-individual variations of wrist abduction (*β*_*w*_) were smaller than intra-individual variances for the buccal area of both the right and left sides. The horizontal shoulder angle (*ϕ*_*s*_) had the largest intra-individual variance during right side brushing, and the wrist flexion angle (*a*_*w*_) had the largest intra-individual variance during left side brushing.

**Table 3:** Inter- and intra-individual variations of joint angle power spectra during molar brushing

		Buccal			Palatal		
	Angle	Estimate (SE)	Intra-individual	Inter-individual	Estimate (SE)	Intra-individual	Inter-individual
Right	*ϕ*_*s*_	101.83 (25.62)	6062.54	3690.28	104.15 (37.31)	3278.30	11,437.44
	*ψ*_*s*_	44.34 (10.22)	416.70	786.32	58.37 (20.12)	193.37	3578.99
	*θ*_*e*_	40.96 (7.00)	137.09	390.68	35.22 (6.70)	69.67	380.51
	*α*_*w*_	120.16 (14.44)	2707.95	891.97	112.37 (17.27)	1476.07	2191.27
	*β*_*w*_	70.35 (10.31)	1043.09	575.53	54.15 (12.16)	453.48	1180.57
Left	*ϕ*_*s*_	99.36 (11.90)	225.67	1194.43	116.56 (21.54)	1849.30	3558.80
	*ψ*_*s*_	50.91 (11.28)	172.13	1083.67	56.00 (10.55)	607.63	799.85
	*θ*_*e*_	45.77 (7.90)	97.38	526.77	50.67 (10.90)	357.85	949.93
	*α*_*w*_	145.35 (21.92)	2646.19	3398.55	143.11 (15.28)	3487.85	937.95
	*β*_*w*_	59.76 (10.29)	1266.89	510.57	57.07 (12.50)	1062.82	1051.33

During palatal brushing, intra-individual variations of both shoulder angles (*ϕ*_*s*_, *ψ*_*s*_) and the elbow flexion angle (*θ*_*e*_) were smaller than their corresponding inter-individual variations when brushing both the right and left sides. The inter-individual variations of both wrist angles (*a*_*w*_, *β*_*w*_) when brushing the left side were smaller than their corresponding intra-individual variations, although the variance for *β*_*w*_ was very small. The horizontal shoulder angle (*ϕ*_*s*_) had the largest intra-individual variance during right side brushing, and the wrist flexion angle (*a*_*w*_) had the largest intra-individual variance during left side brushing. Under all conditions, the inter- and intra-individual variations of the elbow flexion angle (*θ*_*e*_) were smaller than for any of the other angles.

## Discussion

During buccal brushing, individuals showed less inter-individual variation during right side brushing (Table 1). It may be that twisting the wrist toward the palm in the pen grip during buccal right side brushing limited the range of motion and made precise control of brushing motion more difficult. Additionally, the relatively small intra-individual variation during left side brushing of the buccal area showed that the dental hygienists had a stable personal brushing motion for this task. This suggests that brushing the left buccal area might be easier than brushing the right side and would be consistent with reports of greater difficulty in removing dental plaque from upper buccal right teeth than left side teeth [18]. This hypothesis is supported by motion analysis of brushing.

On the other hand, when brushing the palatal area, individual dental hygienist had a more stable brushing motion on the right side rather than the left (Table 1). During right side brushing of the palatal area with a pen grip, the index and middle finger might better control the reciprocating motion of the toothbrush. In contrast, during left side brushing, only the thumb controls the motion, and the stability of brushing motion might decrease. The thumb controls the gripping force when writing with a pen grip [19] while the index and middle fingers control the writing pressure [20]. Power control of tooth brushing with a pen grip might be similar to that of writing.

There were significant differences in the frequency of all joint angles between right and left side brushing of each buccal and palatal area, except for the horizontal shoulder angle (*ϕ*_*s*_) during palatal brushing (Figs. 9 and 10). However, within each brushing area, there were no significant differences among joint angle frequencies. Intra-individual variations of all joint angle frequencies were stable (Table 2), suggesting that each individual had a characteristic rhythm during tooth brushing that was generated synchronously in all arm joints regardless of the brushing location.

Individual characteristic rhythms at various points on the body during walking, hopping, and locomotion help maintain head and postural stability. Latt et al. reported that the best overall stability was achieved with an individual’s usual step length and cadence [9]. Henmi et al. analyzed motion for shampooing and reported that the joints of the neck, shoulder, and elbow moved in coordination, not separately [21]. This study shows the same pattern for tooth brushing that the joints which constitute each bodily part move in cooperation.

Some joint angles had significantly different power spectra between joints in each brushing area. It has been reported that upper and lower limbs controlled joint movement according to the exercise purpose and intensity [22, 23]. Galloway et al. examined the relationship of muscle and interaction torques to joint acceleration at the shoulder, elbow, and wrist during point-to-point arm movements to a range of targets in the horizontal plane and concluded that the dynamics differed between the joints [24]. This, division of roles among joints according to the aim of the motion is important in activities of daily living.

When brushing both the buccal and palatal aspects of the right area, the horizontal shoulder angle (*ϕ*_*s*_) had relatively large intra-individual variation. This indicates the diversity of shoulder motion. The influence of shoulder movement on the motion of the hand and fingers during daily activities and sporting events has been reported. Dai et al. analyzed the relationship between shoulder joint angle and performance in discus throwing and reported that performance was significantly negatively correlated with arm-shoulder separation angle [25]. Dounskaia et al. reported that the shoulder creates a foundation for motion of the entire arm during drawing [26].

On the other hand, when brushing both the buccal and palatal aspects of the left area, wrist flexion angle (*a*_*w*_) had a relatively large intra-individual variation. This indicates the diversity of wrist motion. Hand-arm motion has been analyzed not only during daily activity but also during physiological tremor and hand-arm vibration syndrome. Luker et al. quantitatively evaluated the relationship between hand-arm motion and thumb force and reported strong correlations between them [27]. Thus, the shoulder and wrist are important contributors to the performance of finger and hand brushing motion. However, the reasons for the differences in roles of the shoulder and wrist joints between the right and left side brushing are not clear from the current study. Future studies will be necessary to confirm and explain these differences.

The smaller inter- and intra-individual variations of elbow flexion (*θ*_*e*_) when brushing both the buccal and palatal areas indicate that the motion at the elbow is stable. Dounskaia et al. reported that the elbow serves as a fine tuner of end-point movement, and the arm joints share roles during movement [26]. However, our results suggest that elbow function is important for generating the rhythm of brushing.

In this study, we did not include additional instrumentation to measure brushing force. Attaching other instrumentation to the brush would have changed the weight of the toothbrush, which in turn might have influenced the brushing motion. We gave priority to collection and analysis of the tooth brushing motion data in the most natural state possible, because elucidation of tooth brushing motion was the main purpose in this study.

Recently, Tosaka et al. demonstrated the effectiveness of analyzing tooth brushing cycles using a system that measures tooth brushing motion with an accelerometer and tooth brushing force with a strain tension gage attached to a toothbrush. They reported that tooth brushing motion and brushing force change depending on the brushing location [28]. Their results suggest that it will be necessary to examine the relation between tooth brushing motion and grasp or brushing pressure by using other instrumentation in the future because of the possibility of a close relationship between motion and force.

In this study, the upper molar was selected as the target of analysis, and the brushing motion in the right and left or palatal and buccal directions was considered. Practically, the dental arch can be separated into 12 brushing areas (i.e., upper, lower, right, left, palatal (lingual), buccal (labial), molar, and incisor). To compare differences of brushing motion of each area all at once would be extremely complex because of the wide variety of motion. We therefore analyzed only the upper molar area, although other areas of interest could be considered using the same techniques in future studies.

Our study quantitatively evaluated tooth brushing motion as the individual’s toothbrush frequency, coordinated movement of the joints of the arm, and rhythm generation of brushing. Our results might provide a guide for dental professionals to instruct brushing motion. To instruct adjustments in the proper position of the toothbrush to brush the teeth, the movements of the shoulder when brushing on the right and the wrist when brushing on the left (both buccal and palatal) were important. It might be better to fix their elbow during brushing as a fulcrum of motion because the elbow generates the rhythm of brushing. The frequency of the reciprocation of the toothbrush is 3.5~4.5 Hz.

## Conclusion

We evaluated the frequencies and power spectra of toothbrush motion and joint angles of the shoulder, elbow, and wrist during tooth brushing by a sample of dental hygienists. These results support the following:

1. It was confirmed quantitatively that dental hygienists use individual characteristic rhythms during tooth brushing.
2. All arm joints moved synchronously during brushing, and tooth brushing motion was controlled by coordinated movement of the joints.
3. The elbow generated an individual’s frequency through a stabilizing movement.

## References

[1] Bowden J, Scully C (1989). Dentistry and total oral health. BMJ.

[2] Jahn CA (2004). Good oral health contributes to good total health: the role of the diabetes educator. Diabetes Educ.

[3] Das UM, Singhal P (2009). Tooth brushing skills for the children aged 3–11 years. J Indian Soc Pedod Prev Dent.

[4] Poyato-Ferrera M, Segura-Egea JJ, Bullon-Fernandez P (2003). Comparison of modified bass technique with normal toothbrushing practices for efficacy in supragingival plaque removal. Int J Dent Hyg.

[5] Schlueter N, Klimek J, Saleschke G, Ganss C (2010). Adoption of a toothbrushing technique: a controlled, randomised clinical trial. Clin Oral Investig.

[6] Poche C, McCubbrey H, Munn T (1982). The development of correct toothbrushing technique in preschool children. J Appl Behav Anal.

[7] Kim KS, Yoon TH, Lee JW, Kim DJ (2009). Interactive toothbrushing education by a smart toothbrush system via 3D visualization. Comput Methods Programs Biomed.

[8] Graetz C, Bielfeldt J, Wolff L, Springer C, El-Sayed KM (2013). Toothbrushing education via a smart software visualization system. J Periodontol.

[9] Latt MD, Menz HB, Fung VS, Lord SR (2008). Walking speed, cadence and step length are selected to optimize the stability of head and pelvis accelerations. Exp Brain Res.

[10] Laudani L, Casabona A, Perciavalle V, Macaluso A (2006). Control of head stability during gait initiation in young and older women. J Electromyogr Kinesiol.

[11] Mulavara AP, Verstraete MC, Bloomberg JJ (2002). Modulation of head movement control in humans during treadmill walking. Gait Posture.

[12] Green JR, Wilson EM (2006). Spontaneous facial motility in infancy: a 3D kinematic analysis. Dev Psychobiol.

[13] Hwang S, Kim Y (2009). Lower extremity joint kinetics and lumbar curvature during squat and stoop lifting. BMC Musculoskelet Disord.

[14] Armand M, Minor LB (2001). Relationship between time- and frequency-domain analyses of angular head movements in the squirrel monkey. J Comput Neurosci.

[15] Bardal EM, Roeleveld K, Johansen TO, Mork PJ (2012). Upper limb position control in fibromyalgia. BMC Musculoskelet Disord.

[16] Hingtgen B, McGuire JR, Wang M, Harris GF (2006). An upper extremity kinematic model for evaluation of hemiparetic stroke. J Biomech.

[17] Matsuo K, Hiiemae KM, Palmer JB (2005). Cyclic motion of the soft palate in feeding. J Dent Res.

[18] Lee WCSM, Suzuki J, Takeyama K, Katai H, Nomura T, Arai T, Nakamura J (1986). Plaque control record by O’Leary et al. In initial periodontal treatment. J Japan Soc Periodontol.

[19] Udo H, Otani T, Udo A, Yoshinaga F (2000). An electromyographic study of two different types of ballpoint pens—investigation of a one hour writing operation. Ind Health.

[20] Baur B, Schenk T, Furholzer W, Scheuerecker J, Marquardt C (2006). Modified pen grip in the treatment of writer’s cramp. Hum Mov Sci.

[21] Henmi S, Yonenobu K, Masatomi T, Oda K (2006). A biomechanical study of activities of daily living using neck and upper limbs with an optical three-dimensional motion analysis system. Mod Rheumatol.

[22] Mourey F, Pozzo T, Rouhier-Marcer I, Didier JP (1998). A kinematic comparison between elderly and young subjects standing up from and sitting down in a chair. Age Ageing.

[23] Pozzo T, Berthoz A, Lefort L (1989). Head kinematic during various motor tasks in humans. Prog Brain Res.

[24] Galloway JC, Koshland GF (2002). General coordination of shoulder, elbow and wrist dynamics during multijoint arm movements. Exp Brain Res.

[25] Dai B, Leigh S, Li H, Mercer VS, Yu B (2013). The relationships between technique variability and performance in discus throwing. J Sports Sci.

[26] Dounskaia N, Ketcham CJ, Stelmach GE (2002). Commonalities and differences in control of various drawing movements. Exp Brain Res.

[27] Luker KR, Aguinaldo A, Kenney D, Cahill-Rowley K, Ladd AL (2014). Functional task kinematics of the thumb carpometacarpal joint. Clin Orthop Relat Res.

[28] Tosaka Y, Nakakura-Ohshima K, Murakami N, Ishii R, Saitoh I (2014). Analysis of tooth brushing cycles. Clin Oral Investig.

